# Neuromodulation techniques targeting neurotransmitter dysfunction: Innovation in treatments for Alzheimer’s disease

**DOI:** 10.4103/NRR.NRR-D-25-00582

**Published:** 2025-10-30

**Authors:** Siyuan Jin, Bingqi Guo, Wensi Hao, Xin Su, Tingting Zhang, Chunyan Liu

**Affiliations:** 1 Department of Neurology, Xuanwu Hospital, Capital Medical University, Beijing, China; 2 Beijing Key Laboratory of Neuromodulation, Beijing, China

**Keywords:** Alzheimer’s disease, deep brain stimulation, default mode network, neurology, neuromodulation technologies, neurotransmitter agents, transcranial direct current stimulation, transcranial magnetic stimulation, transcranial ultrasound stimulation, vagus nerve stimulation

## Abstract

Alzheimer’s disease is a neurodegenerative disorder characterized by progressive cognitive decline, synaptic dysfunction, and neurotransmitter imbalance. Novel neuromodulation approaches, both non-invasive and invasive, show promising potential for restoring neural circuit integrity and improving cognition. This review aims to elucidate the relationship between neuromodulation, neural network circuits, and neurotransmitters. It systematically synthesizes recent advances in neuromodulation techniques, focusing on their ability to modulate four critical neurotransmitter systems: cholinergic, glutamatergic, GABAergic, and monoaminergic systems. Additionally, this review establishes a critical association between neurotransmitter regulation and synaptic plasticity mechanisms, proposing a novel “circuit-transmitter” triad framework for intervention. It represents the first systematic integration of the neurotransmitter regulation mechanisms of various neuromodulation techniques while evaluating their clinical viability for Alzheimer’s disease intervention. Beyond established targets, this review identifies the hippocampus-thalamus axis, linked via direct entorhinal-thalamic projections, as a promising focus for ultrasonic neuromodulation research.

**Facts**

• Pathological features of Alzheimer’s disease: The pathological features of Alzheimer’s disease include amyloid-β plaques, tau protein tangles, and neuronal loss. These factors lead to disruptions in the reorganization of large-scale neural networks, thereby affecting cognitive function.

• Role of neurotransmitter systems: Dysregulation of neurotransmitter systems, such as acetylcholine, glutamate, γ-aminobutyric acid, norepinephrine, serotonin, and dopamine, is a key factor in the pathological process of Alzheimer’s disease, affecting synaptic transmission and the dynamic balance of neural networks.

• Potential of neuromodulation techniques: Neuromodulation techniques, such as transcranial magnetic stimulation, transcranial direct current stimulation, transcranial ultrasound stimulation, and deep brain stimulation, show potential for improving cognitive function in patients with Alzheimer’s disease by modulating the activity of specific brain regions and neurotransmitter levels.

**Open questions**

• What are the mechanisms of action of neurotransmitters associated with Alzheimer’s disease?

• What are the mechanisms of action of neuromodulation techniques related to Alzheimer’s disease?

• What are the pathological mechanisms associated with Alzheimer’s disease and the related changes in brain networks?

## Introduction

Alzheimer’s disease (AD) is a progressive neurodegenerative disorder that progressively damages the central nervous system through ongoing neurodegeneration (Asimakidou et al., 2025; Ji et al., 2025), leading to cognitive decline and behavioral deficits (Weller and Budson, 2018). It manifests as a spectrum of neuropsychological impairments, including memory dysfunction, aphasia, apraxic motor planning difficulties, agnosia, visuospatial disorientation, compromised abstract reasoning and computational abilities, as well as changes in personality and behavioral disturbances. These multidimensional deficits collectively reflect the pathophysiological disruption of cortical and hippocampal neural networks (Pini et al., 2016). These epidemic places catastrophic socioeconomic burdens on healthcare systems and caregivers, with epidemiological models projecting that cases will double by 2050 due to an aging population (Weller and Budson, 2018). Global prevalence of dementia is expected to increase from 50 million cases in 2010 to 113 million by 2050 (Knopman et al., 2021). Without effective interventions to stop disease progression, the number oAD cases could reach 13.8 million by 2060 (2023 Alzheimer’s Disease Facts and Figures, 2023). This accelerating trend indicates AD as a critical public health challenge, highlighting the urgent need for age-stratified preventive strategies and neuroprotective interventions. AD is characterized by progressive memory loss, synaptic dysfunction, and neuronal death; its pathogenesis remains multifactorial and elusive (Cai et al., 2025; Figueiredo-Pereira et al., 2025; Tinu et al., 2025). Dominant hypotheses implicate amyloid-beta (Aβ) plaques, tau neurofibrillary tangles, and cholinergic deficits as central drivers of neurodegeneration (Sharma, 2019).

Current diagnostic frameworks for AD identify three core categories of biomarkers, as defined by the AT(N) classification system: Aβ pathology, which can be detected using the cerebrospinal fluid (CSF) Aβ_42/40_ ratio or amyloid-positron emission tomography (PET) imaging; hyperphosphorylated tau protein accumulation, measurable through CSF phosphorylated tau (p-tau) 181/217 or tau-PET tracers; and neurodegeneration biomarkers, which include CSF total tau, plasma neurofilament light chain (NfL), and quantification of hippocampal atrophy through magnetic resonance imaging (MRI) (Jack et al., 2024). Together, these biomarkers facilitate the *in vivo* characterization of AD neuropathological processes, in accordance with the 2018 NIA-AA research criteria for accurate disease staging and validation of therapeutic targets (Jack et al., 2024).

Accessible blood–based biomarkers are democratizing the diagnosis of AD, promoting equitable healthcare delivery across underserved populations and resource-constrained settings. This transformation is physiologically rooted in the translocation of neuropathological proteins through compromised blood–brain barrier (BBB). During BBB dysfunction, a hallmark of neurodegeneration, CSF-derived Aβ isoforms and p-tau species enter systemic circulation through disrupted tight junctions and increased paracellular flux (Cai et al., 2018). While minimally invasive biomarkers for AD show promising diagnostic potential, their clinical validation presents significant challenges. Neurodegeneration indicators such as NfL and t-tau exhibit etiological promiscuity, increasing non-specifically in conditions such as demyelinating disorders, neurodegenerative processes, cerebrovascular incidents, and traumatic encephalopathy. As a result, these markers lack sufficient specificity for definitive identification of AD neuropathology without complementary diagnostics (Arslan and Zetterberg, 2023).

The main pharmacological treatment options for AD include acetylcholinesterase (AChE) inhibitors, N-methyl-D-aspartate (NMDA) receptor antagonists, and various therapies targeting Aβ and tau. While these agents temporarily improve cognitive symptoms by modulating cholinergic transmission or reducing glutamate excitotoxicity, they do not address the underlying pathophysiology of Aβ deposition, tau hyperphosphorylation, and neurodegenerative processes (Scheltens et al., 2021). This limitation highlights the critical unmet need for disease-modifying therapies that can halt or reverse the progression of AD, as emphasized in the 2023 NIA-AA therapeutic guidelines (Jack et al., 2024). The limitations of drug therapies include poor BBB penetration, systemic side effects, and the risks associated with medication discontinuation, such as constipation, headaches, and worsening cognitive impairment. Most clinical evidence suggests that metformin, a first-line glucose-lowering medication, may lower the risk of AD among individuals with type 2 diabetes mellitus (Liao et al., 2021). Studies have shown that metformin decreases levels of p-tau in the hippocampus and cortex of mice with scopolamine-induced cognitive impairment (Mostafa et al., 2016) and reduces beta-site amyloid precursor protein cleaving enzyme 1 expression and amyloid plaque accumulation in the hippocampus and cortex of amyloid precursor protein/presenilin 1 (APP/PS1) mice treated with metformin (Ou et al., 2018). However, Ning et al. (2022) concluded that while metformin has shown beneficial effects in AD animal models, current evidence does not support its use in human subjects with mild cognitive impairment or mild AD. The recent regulatory approval of anti-amyloid monoclonal antibodies, such as lecanemab and donanemab, has introduced a new therapeutic paradigm targeting the amyloid cascade in AD. However, we must recognize that the clinical benefits of these monoclonal antibodies in slowing cognitive decline may not outweigh the significant risks of serious treatment-related adverse events. Therefore, the safety profile and clinical efficacy of these interventions require thorough evaluation before clinical application, while this study suggests novel therapeutic avenues.

**Figure 1:**
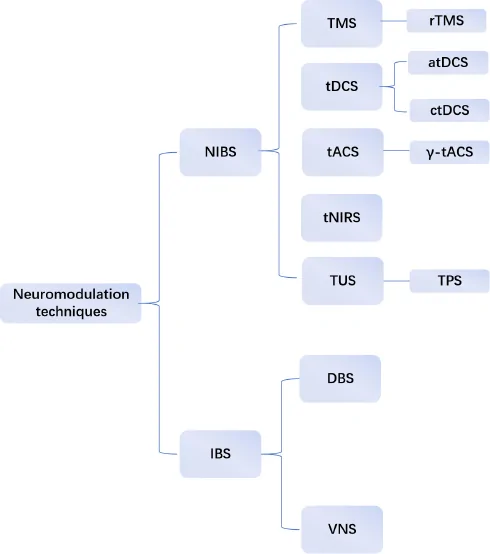
Currently available neuromodulation techniques. atDCS: Anodal tDCS; ctDCS: cathodal tDCS; DBS: deep brain stimulation; IBS: invasive brain stimulation; NIBS: non-invasive brain stimulation; tACS: transcranial alternating current stimulation; tDCS: transcranial direct current stimulation; TMS: transcranial magnetic stimulation; TPS: transcranial pluse stimulation; tNIRS: transcranial near-infrared light stimulation; TUS: transcranial ultrasound stimulation; VNS: vagus nerve stimulation.

Non-pharmacological neuromodulation techniques have recently emerged as important tools to address these challenges (Koch et al., 2024). These techniques (**Figure 1**), including noninvasive repetitive transcranical magnetic stimuiation (rTMS), repetitive transcranial electrical stimulation encompassing transcranial direct current stimulation (tDCS) and transcranial alternating current stimulation (tACS), transcranial ultrasound stimulation (TUS), transcranial near-infrared light stimulation (tNIRS), as well as invasive deep brain stimulation (DBS) and vagus nerve stimulation (VNS), have shown clinical effectiveness in improving cognitive function and regulating neural transmission (Koch et al., 2024). For example, rTMS enhances cortical plasticity by regulating glutamatergic signaling (Cao et al., 2022), while tDCS rebalances excitatory/inhibitory (E/I) ratios by modulating γ-aminobutyric acid (GABA) and glutamate levels (Lengu et al., 2021). Non-invasive brain stimulation (NIBS) has emerged as a pivotal therapeutic modality for directly modulating cortical activity through the bidirectional regulation of cortical excitability, offering a non-pharmacological approach to intervening in neurodegenerative pathologies (Pini et al., 2022). These stimulation techniques enable precise targeting of circuits vulnerable to AD, such as the default mode network (DMN) and septo-hippocampal pathways. However, the molecular mechanisms of these technologies in treating AD are not yet clear. This review synthesizes cutting-edge evidence on how neuromodulation techniques intervene in neurotransmitter systems—acetylcholine (ACh), glutamate, GABA, and monoamines—to restore synaptic homeostasis and cognitive function. By bridging molecular mechanisms with clinical outcomes, we highlight the transformative potential of these tools to shift AD treatment from symptomatic management to disease modification.

**Figure 2:**
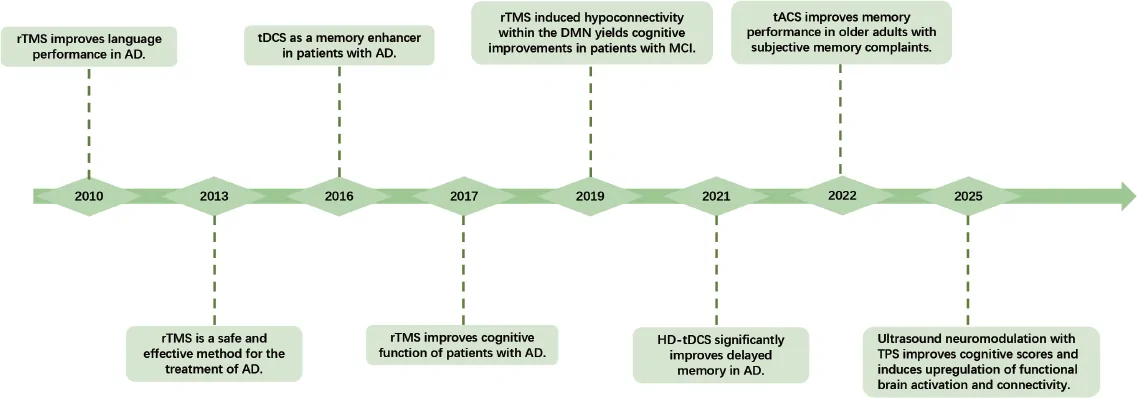
Non-invasive neuromodulation techniques enhancing cognitive function in patients with AD. AD: Alzheimer’s disease; DMN: default mode network; HD-tDCS: high-definition transcranial direct current stimulation; MCI: mild cognitive impairment; rTMS: repetitive transcranial magnetic stimulation; tACS: transcranial alternating current stimulation; tDCS: transcranial direct current stimulation; TMS: transcranial magnetic stimulation; TPS: transcranial pulse stimulation.

Current neuromodulation techniques have demonstrated partial efficacy in ameliorating cognitive deficits in AD; however, no comprehensive review systematically synthesizes neurotransmitter-targeted intervention strategies (**Figure 2**). This review specifically focuses on neurotransmitter systems as the mechanistic foundation, critically evaluating the therapeutic potential of contemporary neuromodulation approaches in modulating cholinergic, glutamatergic, GABAergic, and monoaminergic pathways. We elucidate how these techniques can improve cognitive function and hope that non-pharmacological neuromodulation targeting neurotransmitter systems may offer new treatments for AD.

## Search Strategy

A comprehensive literature search was conducted to identify all studies involving neuromodulation techniques for AD, alterations in AD-related neurotransmitters, and network changes. Databases, including PubMed and Web of Science, were searched from their inception to May 15, 2025. The search incorporated MeSH terms and free-text terms such as “repetitive transcranial magnetic stimulation,” “Alzheimer’s Disease,” “Neurology,” “Neurotransmitter Agents,” “Neuromodulation Techniques,” “Default Mode Network,” “Transcranial Magnetic Stimulation,” “Transcranial Direct Current Stimulation,” “Transcranial Ultrasound Stimulation,” “Deep Brain Stimulation,” “Vagus Nerve Stimulation,” and their synonyms. A total of 180 articles were included. Given the limited research on neuromodulation-induced neurotransmitter alterations in patients with AD, we considered including all clinical trials in this review. Inclusion criteria comprised clinical trials, articles published in English or Chinese, studies involving patients with AD aged ≥ 18 years diagnosed by cognitive neurology experts according to the International Classification of Diseases, 10^th^ Revision (ICD-10, code F00) (DiSantostefano, 2009), and investigations exploring neuromodulation applications. Exclusion criteria included irrelevant studies, duplicate publications, editorial materials, patents, and conference abstracts.

## Pathophysiological Basis of Alzheimer’s Disease

### Pathological features of Alzheimer’s disease

AD presents macroscopically with significant cerebral atrophy, accompanied by decreased brain weight, widened sulci, narrowed gyri, and conspicuous shrinkage affecting the temporal lobe—most notably the hippocampus, which exhibits volume loss exceeding 40% in advanced Braak stages. At the histopathological level, AD is defined by extracellular neuritic plaques consisting of Aβ deposits, intraneuronal neurofibrillary tangles formed by hyperphosphorylated tau proteins, selective neuronal loss, and reactive gliosis characterized by hypertrophic astrocytes along with activated microglia clustered around amyloid plaques (Terry et al., 1991).

AD neuropathology is characterized by the presence of Aβ plaques and activated glial cells that display pro-inflammatory phenotypes, both of which can be detected using molecular imaging techniques (Knopman et al., 2021). Pathologically, hyperphosphorylation of tau at specific epitopes induces conformational changes that promote its aggregation into paired helical filaments, which accumulate in somatodendritic compartments. This aggregation process contributes to the progression of AD by disrupting synaptic function and impairing neural network connectivity (Congdon et al., 2023). Simultaneously, neurotoxic Aβ_42_ isoforms emerge through the amyloidogenic processing pathway, wherein β-secretase and γ-secretase sequentially cleave the APP, a synaptic transmembrane scaffold, resulting in the production of fibrillogenic peptides. While Aβ_42_ exhibits a high propensity for fibril formation, non-amyloidogenic APP processing yields neuroprotective fragments such as APPsα. This fragment modulates synaptic transmission by interacting with GABA_A_ receptor subunits, indicating that pharmacological enhancement of GABAergic signaling may be a therapeutic strategy to mitigate cognitive deficits associated with AD (Bi et al., 2025).

Neuroanatomically, the entorhinal cortex—recognized as the earliest region exhibiting neurofibrillary tangle accumulation in AD—maintains strong structural connectivity with the temporoparietal and prefrontal association cortices via the perforant pathway. This neural circuitry is replicated in healthy individuals through resting-state functional magnetic resonance imaging (fMRI) but is markedly disrupted in patients with symptomatic AD, reflecting profound network disintegration (Igarashi, 2023). An expanding corpus of empirical research substantiates the pivotal role of the spatiotemporal interaction among Aβ plaque deposition, neurofibrillary tau tangle formation, and macroscale neural network restructuring within the AD pathogenic cascade. This complex triad constitutes a fundamental pathogenic axis, wherein the progressive disconnection of critical hub regions within these networks is increasingly recognized as a key mechanism driving both the gradual cognitive decline and the heterogeneous neuropsychiatric manifestations characteristic of AD.

### Large-scale neural network reorganization in Alzheimer’s disease is impaired

The development of human cognition fundamentally relies on the spatiotemporal integration of large-scale neural networks—specifically the DMN, the salience network (SN), and central executive network (CEN)—along with limbic system structures, including the Papez circuit. This integration is facilitated by dynamic interactions mediated through synchronized oscillatory activity within the theta (4–8 Hz) and gamma (30–100 Hz) frequency bands (Ibrahim et al., 2021). Such multidimensional convergence is orchestrated through hierarchical neural transmission pathways: the DMN supports self-referential cognitive processes by exchanging information between the posterior cingulate cortex (PCC) and the hippocampus; the SN dynamically modulates attentional resource allocation through coherence between the anterior insula and the dorsal anterior cingulate cortex (ACC); and the CEN regulates working memory functions through interactions within the dorsolateral prefrontal-parietal circuitry. Concurrently, the Papez circuit integrates affective valence with episodic memory through synaptic signaling along the fornix and mammillothalamic tract. Collectively, these interconnected systems constitute the neurobiological framework underlying cognitive processing (Cheyuo et al., 2022).

The DMN, characterized as a hierarchically structured neurocognitive system, integrates key cortical hubs such as the medial prefrontal cortex (mPFC), PCC, inferior parietal lobule (IPL), and medial temporal lobe (MTL) regions, including the hippocampal and parahippocampal formations. This distributed network supports higher-order cognitive functions through specialized neurocomputational gradients: interactions between the ventromedial prefrontal cortex (PFC) and amygdala facilitate mPFC-mediated self-referential processing and value-based decision-making; dynamic modulation of thalamocortical oscillatory weights enables PCC-dependent multisensory integration; cross-frequency coupling between the angular gyrus and hippocampus underlies IPL-supported episodic memory consolidation; and mechanisms involving dentate gyrus-CA3 circuitry and anterior hippocampal future projection contribute to MTL-driven allocentric spatial mapping and mental chronometry (Menon, 2023). A resting-state fMRI study further corroborates these observations, revealing significantly diminished DMN coherence in TgF344-AD transgenic rat models compared to wild-type controls (Tudela et al., 2019).

The anterior insula and ACC collectively constitute the SN, which operates as a specialized neurocomputational architecture designed to detect environmental salience and hierarchically prioritize behaviorally significant stimuli across interoceptive bodily states and exteroceptive environmental contexts, thereby coordinating adaptive neurobehavioral responses (Menon and Uddin, 2010). This system critically underpins social cognition, interpersonal affect sharing, and self-referential awareness through its sophisticated integration of emotionally valenced sensory inputs, cognitive representations, and visceral feedback signals (Chand et al., 2017). During goal-directed tasks, the SN exhibits dynamic network reconfiguration, characterized by strengthened frontoparietal network (FPN) coupling concomitant with DMN decoupling, which facilitates attentional resource allocation toward external demands. Conversely, in resting states, it undergoes a phase transition marked by FPN disengagement and DMN resynchronization (Chand et al., 2017). Anatomically, the SN demonstrates a tripartite neurobiological stratification: its dorsal stream mediates dynamic attentional shifts and cognitive control through dorsolateral prefrontal-cingulate projections, while the ventral axis orchestrates affective processing via insulo-amygdalar pathways. The intermediate opercular sector integrates viscerosomatic-affective signals through bidirectional parabrachial nucleus connectivity, collectively forming a metastable system for environmental contingency monitoring. A pioneering neurophysiological inquiry systematically analyzed intracranial electroencephalographic recordings across 177 human subjects engaged in four discrete episodic memory paradigms, each implementing rigorously controlled encoding-retrieval cycles. Phase-amplitude coupling-directed transfer entropy quantification demonstrated statistically robust directional information flux emanating from the dorsal anterior insula—a pivotal SN hub—propagating toward cardinal nodes within both the DMN and FPN networks (Das and Menon, 2024). Crucially, the SN maintained sustained gamma-band oscillatory engagement throughout the entire mnemonic processing continuum—from task onset to behavioral execution—indicating its overarching regulatory role in memory functions. This network exhibited context-contingent functional reconfiguration: during retrieval epochs, it established theta-range phase-synchronized coupling with the DMN, whereas perceptual encoding phases evoked beta-band coherence with the dorsal attention network (DAN). Collectively, these electrophysiological patterns suggest that the SN operates as a domain-general, metastable control system that dynamically coordinates large-scale network interactions during episodic memory through adaptive re-entrant looping mechanisms, functioning as a polymorphic hub capable of millisecond-scale reconfiguration to meet evolving cognitive demands (Das and Menon, 2024).

The DAN and the DMN exhibit a dynamic interplay characterized by reciprocal inhibition, particularly evident during goal-directed tasks. When the DMN is downregulated, which occurs during focused attention and external task engagement, the DAN becomes active, facilitating the allocation of attentional resources towards exogenous stimuli. This interaction is critical for maintaining cognitive efficiency, as it allows the brain to suppress internally-focused thoughts, such as autobiographical memories, in favor of processing external information. At a neurochemical level, this antagonistic behavior is supported by glutamatergic thalamocortical pathways and noradrenergic inputs from the locus coeruleus. These inputs play a pivotal role in prioritizing cortical activity: the DAN promotes alpha-band desynchronization (8–12 Hz) in areas like the superior parietal lobule, which enhances attentional focus, while simultaneously inhibiting delta-theta oscillations (1–7 Hz) in the precuneus, a region associated with DMN activity, through GABAergic mechanisms. This finely-tuned neurochemical modulation is essential for achieving a state of metastable equilibrium, which enables quick cognitive transitions. The phasic release of norepinephrine selectively boosts the signal-to-noise ratio in the DAN pathways during sensory processing, enhancing focus on relevant stimuli. Concurrently, norepinephrine promotes DMN suppression by hyperpolarizing posterior cingulate neurons via α2-adrenoreceptor activation, further facilitating the shift from internal to external cognitive processes. This balance between the DAN and DMN is crucial for effective cognitive performance, allowing for flexible adaptation to changing task demands.

### Interaction between amyloid-β plaques and large-scale neural network reorganization impairment in patients with Alzheimer’s disease

A multimodal neuroimaging study reveals that individuals carrying the apolipoprotein E (APOE) ε4 allele, a key genetic risk factor for sporadic AD, display unique changes in brain network organization (Mentink et al., 2021). These changes include a breakdown of the DMN and increased connectivity within limbic circuits, even during the preclinical stage of subjective cognitive decline before the Aβ accumulation is detectable. These APOE ε4-associated connectomic signatures, characterized by lower overall network efficiency and increased nodal centrality in the PCC, demonstrate predictive validity for subsequent cognitive deterioration independent of amyloid level. This suggests that genetic risk modulates brain network resilience through processes related to lipid metabolism disruption and synaptic instability (Brier et al., 2014).

Aβ accumulation occurs in a network-specific manner, mainly within the DMN. A PET study shows that individuals who will develop Aβ pathology display increased DMN connectivity and higher glucose metabolism before symptoms appear (Palop and Mucke, 2016). This aberrant synaptic hyperactivity leads to Aβ overproduction through activity-dependent APP cleavage, creating a vicious cycle where disrupted homeostasis in highly active DMN regions, such as the PCC, causes trans-synaptic network failure in structurally connected areas such as the MTL (Buckner et al., 2009). The PCC, a pivotal hub of early synaptic dysfunction and Aβ accrual, exhibits strong structural and functional coupling with the MTL through bidirectional glutamatergic and GABAergic pathways (Minoshima et al., 1997). Within the DMN, chronic synaptic dysregulation—manifested as abnormal neuronal hyperexcitability and impaired synaptic plasticity—drives the gradual accumulation of Aβ oligomers over many years. This persistent synaptic stress spread trans-synaptically through highly interconnected DMN modules, promoting the hierarchical spread of tau pathology via prion-like templated misfolding (Simic et al., 2014).

This spatial and temporal pattern follows principles of brain connectivity: Aβ oligomers propagate trans-synaptically along glutamatergic pathways via activity-dependent mechanisms, while tau neurofibrillary tangles spread through axonal transport routes defined by structural connectome topology (Vogel et al., 2020). In the preclinical stages of AD, fibrillar Aβ deposits mainly affect DMN nodes, leading to early disruption in the functional coupling between the hippocampus and precuneus, as well as abnormal decoupling of the DMN from DAN, a pattern of functional disconnection that can be detected decades before clinical symptoms appear (Ward et al., 2015).

Reduced connectivity is primarily found in the PCC and precuneus—central hubs of the DMN that show a particular susceptibility to the buildup of Aβ oligomers (Braak stage II-III) and the spread of tau in the transentorhinal region (Pievani et al., 2017).

The MTL plays a crucial role in memory encoding driven by novelty. Both electrophysiological and functional neuroimaging studies consistently show sustained activation across MTL regions—particularly in the anterior hippocampus—when processing new stimuli during declarative memory tasks. This neural response typically diminishes with repeated exposure to the same stimulus, reflecting an inverse relationship with familiarity (Kumaran and Maguire, 2006). The mechanism for novelty detection is established through hippocampal-entorhinal circuits, where the presentation of unexpected stimuli elicits theta-gamma phase-amplitude coupling (4–8 Hz theta oscillations nested within 30–100 Hz gamma bursts) in the anterior hippocampus. This activity is dynamically adjusted based on the frequency of stimulus recurrence and is disrupted by hippocampal damage, which specifically impairs the memory benefits associated with novelty (Bastin et al., 2019). Furthermore, the connection between the PCC and MTL serves as a key hub for the cross-seeding of Aβ and tau proteins, thereby promoting the progression of neurodegenerative processes. In this pathway, Aβ-related network instability enhances tau phosphorylation and misplacement within dendrites, ultimately leading to the breakdown of large-scale cognitive networks.

In a typical brain, the DMN and SN interact in a dynamic, opposing manner regulated by competitive glutamate signaling and noradrenaline modulation. When the SN, located in the dorsal anterior cingulate and anterior insula, activates to focus on external stimuli, the DMN, centered in the posterior cingulate/PC and mPFC, becomes less active due to GABA-mediated inhibition of its key areas, and *vice versa* (Pini et al., 2022). This opposing functional relationship, maintained by the coupling of SN-driven gamma waves and DMN-related alpha rhythms, breaks down in AD. In AD, the DMN shows reduced connectivity with disrupted theta-gamma interactions, while the SN becomes overly synchronized, exhibiting abnormally sustained beta-band activity (15–30 Hz) in the anterior insula and striatal circuits. This imbalance impairs the brain’s ability to switch between networks, resulting in persistent SN dominance that leads to excessive attention to irrelevant stimuli and weakens DMN-related memory processes. This pathological state is exacerbated by Aβ-induced disturbances in noradrenaline regulation from the locus coeruleus (LC) and reduced GABA inhibition in the thalamic reticular nucleus (Pini et al., 2022).

## Neurotransmitter Dysfunction in Alzheimer’s Disease: From Pathogenesis to Therapeutic Targets

Neurotransmitter systems are foundational to neural communication, orchestrating processes that range from basic synaptic transmission to complex cognitive functions. In AD, the accumulation of Aβ plaques, tau tangles, and neuroinflammation collectively disrupts these systems, leading to synaptic dysfunction and circuit-level impairments. This section reviews the molecular, cellular, and circuit-level roles of neurotransmitters such as ACh, glutamate, GABA, and monoamines in the progression of AD, while highlighting new therapeutic approaches aimed at modulating neurotransmitter activity.

**Acetylcholine**

The cholinergic hypothesis is the earliest theory regarding the pathogenesis of AD. Ach is a pivotal neurotransmitter involved in learning, memory, sleep, and other behaviors. ACh is synthesized in cholinergic neurons from choline and acetyl-CoA by choline acetyltransferase (ChAT). It is released into the synaptic cleft, where it binds to nicotinic and muscarinic receptors, affecting synaptic transmission and cognition. ACh is degraded by enzyme AChE in the synaptic cleft, and choline is reabsorbed by presynaptic neurons for reuse (Parsons et al., 1993). The cholinergic system primarily originates from the basal forebrain nuclei (e.g., medial septum and nucleus basalis of Meynert) and projects extensively to the hippocampus, subiculum, cornu ammonis regions, entorhinal cortex, and cingulate cortex (Yang et al., 2023).

Patients with AD typically exhibit degeneration of deep and early basal forebrain cholinergic neurons, leading to a severe deficiency of ACh and decreased ACh transferase activity. The degree of dementia is correlated with synaptic loss between the basal forebrain and the target tissues of the hippocampus and cortex, which is considered the main cause of memory decline (Yoon et al., 2013). Hippocampal θ oscillations mediate spatial navigation, environmental encoding, and memory formation. In AD, the degeneration of hippocampal cholinergic projections leads to abnormalities in θ oscillations, impairing memory function (Zhang et al., 2010). The nicotinic ACh receptor (nAChR) family is a prototypical member of the pentameric ligand-gated ion channel. Low nAChR activity has a causal role in the dysregulation of the DMN observed in conditions such as mild cognitive impairment and AD (Zhang et al., 2010). The abnormal cholinergic system also regulates and promotes changes in APP metabolism and tau phosphorylation, leading to neurotoxicity, neuroinflammation, and neuronal death (Majdi et al., 2020). Thus, the ACh system is not only a driving factor of symptoms but also a key participant in the pathological progression of AD.

AChE is the primary enzyme responsible for clearing ACh. AChE inhibitors (e.g., donepezil) are effective therapeutic drugs that temporarily elevate synaptic ACh levels, improving attention and episodic memory in individuals with mild-to-moderate AD (Hampel et al., 2018). However, their diffuse action fails to reverse region-specific neurodegeneration, which may partly explain why the effects of these drugs are typically limited, vary between individuals, and generally serve to maintain cognitive function for an average of only 2 years (Stanciu et al., 2019). Additionally, intraparenchymal delivery of nerve growth factor has shown promise in rescuing cholinergic neurons in Phase I trials; however, challenges such as poor BBB penetrance and off-target glial activation limit clinical translation (Tuszynski et al., 2005).

### Glutamate

Glutamate, the principal excitatory neurotransmitter in the central nervous system, plays a critical role in synaptic plasticity, learning, and memory. It is synthesized from glucose via glycolysis and the hexose monophosphate pathway, and is stored in synaptic vesicles. Upon excitatory stimulation of neurons, glutamate is released into the synaptic cleft and binds to receptors, including α-amino-3-hydroxy-5-methyl-4-isoxazolepropionic acid receptors and NMDA receptors, facilitating information transmission (Yang et al., 2023). Its extracellular levels are tightly regulated by astrocytic transporters, such as glutamate transporter-1. Clinical symptoms in patients with AD are highly correlated with the loss of glutamatergic synapses observed during neuropathological examinations, rather than with amyloid plaque burden or neurofibrillary tangles. Glutamate serves a dual role as a mediator of synaptic plasticity and neurodegeneration in AD (Cheng et al., 2021). Aβ and tau pathologies synergistically disrupt glutamatergic homeostasis, driving excitotoxic neuronal damage and cognitive decline. Aβ oligomers impair astrocytic glutamate uptake, leading to synaptic spillover and persistent activation of extra-synaptic NMDA receptors, which results in calcium overload, mitochondrial dysfunction, and caspase-3-mediated apoptosis (Huang et al., 2017). Additionally, Aβ binds to GluA2 subunits of AMPA receptors, promoting their endocytosis and weakening excitatory synaptic transmission in hippocampal CA1 neurons, a hallmark of early AD (Yeung et al., 2020). Regarding circuit degeneration, glutamatergic projections from the medial septum/diagonal band of Broca (MS/DBB) to the hippocampus (CA1, CA3, and dentate gyrus) are significantly diminished, impairing theta rhythm synchronization and spatial memory encoding (Colom et al., 2005). Drugs targeting NMDA receptors are commonly used in clinical AD treatment. Memantine, a low-affinity non-competitive antagonist of NMDA receptors, reduces excitotoxicity while preserving normal synaptic activity, thereby modestly slowing cognitive decline in moderate-to-severe AD (Bukkeet al., 2020). Combination therapies (e.g., memantine with anti-Aβ antibodies) to synergistically counteract glutamatergic excitotoxicity have also been used (Kim et al., 2024). Additionally, the modulation of the glutamatergic pathway through optogenetic therapy in the mPFC of rodents increased recognition memory (Benn et al., 2016). Due to the bidirectional role of glutamate and its receptors in AD, emerging approaches emphasize precision modulation of E/I balance. Challenges remain in avoiding off-target effects and identifying biomarkers (e.g., MRS-based glutamate/glutamine ratios) to guide personalized treatment.

### γ-Aminobutyric acid

γ-Aminobutyric acid (GABA), the principal inhibitory neurotransmitter in the central nervous system, is synthesized from glutamate via glutamate decarboxylase and stored in synaptic vesicles through the GABA transporter (Engin et al., 2018). Its release depends on presynaptic depolarization and Ca^2+^ influx, with subsequent reuptake by neurons or metabolism by astrocytes to terminate signaling (Yang et al., 2023). GABA exerts its effects through ionotropic GABA_A_ receptors (mediating fast inhibition) and metabotropic GABAB receptors (modulating slow synaptic transmission), collectively regulating neural circuit excitability and synchronizing oscillatory activity critical for memory and cognition.

Disruptions in the GABAergic system, including lower GABA levels, alterations in GABAergic receptors and transporters, and dysfunction of glutamic acid decarboxylase activity, have been observed in patients with AD, contributing to the E/I imbalance during the progression of the disease (Colom et al., 2005). GABAergic reduction has been found in the parietal, temporal, and frontal regions, as well as the hippocampus, which are classically vulnerable to Aβ and tau pathologies. Additionally, reductions have been noted in atypical regions in AD, such as the visual cortex, caudate nucleus, and cerebellum (Villette et al., 2010; Yang et al., 2023). The loss of GABAergic inhibition disrupts theta-gamma coupling, a mechanism underlying working memory and attention, while hyperexcitability resulting from the E/I imbalance accelerates neurodegeneration through excitotoxic pathways (Villette et al., 2010). In patients with AD and transgenic models (e.g., hAPP-J20 mice), GABAergic projections from the MS/DBB to the hippocampal subiculum and CA1 regions are significantly reduced, disrupting the theta and gamma oscillations essential for spatial memory (Rubio et al., 2012).

GABAergic dysfunction in AD reflects a vicious cycle of inhibitory neuron loss, receptor dysregulation, and the collapse of oscillatory networks. However, current therapies targeting the GABAergic system are still lacking. Optogenetic stimulation of MS/DBB GABAergic neurons restores theta oscillations and memory in a mouse model of AD, highlighting neuromodulation as a potential strategy (Rubio et al., 2012).

### Adrenergic system

The adrenergic system, primarily mediated by norepinephrine (NE), plays a critical role in modulating arousal, attention, learning, memory, and emotional regulation. NE is synthesized in neurons, released into the synaptic cleft, and reabsorbed by the NE transporter into presynaptic neurons and glial cells. It is then metabolized by monoamine oxidase (MAO) and catechol-O-methyltransferase. The LC, the primary source of noradrenergic projection neurons, projects to various regions of the brain, including the mPFC, hippocampus (CA1, CA3, dentate gyrus), ACC, thalamus, limbic system, and brainstem, all of which are critical for memory consolidation and executive function (Oleskevich et al., 1989; Lipski and Grace, 2013). A study has found that LC noradrenergic neurons can be significantly degenerated in the brains of patients with advanced AD (Gnegy, 2000). A 30% loss of LC neurons was observed during the transition from a state of no cognitive impairment to amnestic mild cognitive impairment, with an additional 25% loss during the progression to AD (Kelly et al., 2017).

β-Adrenergic receptors (βARs) may be altered by Aβ oligomers. The direct application of human Aβ oligomers can induce the internalization of transfected human beta-2 adrenergic receptors (β2ARs) in fibroblasts and endogenous β2ARs in rodent prefrontal cortical neurons (Wang et al., 2011). Low molecular-weight Aβ oligomers can decrease the levels of β2ARs in neurons, impair hippocampal LTP, and activate microglia *in vivo* (Yang et al., 2017). In terms of circuit deficits, innervation from the LC to the hippocampus decreased by about 69.1% in 5XFAD mice compared to wild-type mice (Jeon et al., 2018). Cognitive impairment in AD may be attributed to the loss of the LC–mPFC pathway, which accounts for more than half of the LC projections to target regions (Kaushal et al., 2016).

Therapies that preserve noradrenergic signaling may offer cognitive benefits and alleviate neuropsychiatric symptoms. The β2 adrenergic agonist clenbuterol and the β1AR agonist xamoterol have been shown to improve memory deficits and reduce social recognition deficits in a mouse model of AD, suggesting that the activation of NE transmission might be effective in treating AD (Chai et al., 2016). However, these drugs have not demonstrated any improvement in AD symptoms in clinical trials, nor have they been applied in clinical practice. While the LC pathway is important in the pathogenesis of AD, no studies have investigated whether changes in the neural circuits originating from the LC can be directly associated with AD-related cognitive and behavioral dysfunction.

### Serotonin and dopamine

Serotonin (5-HT) is synthesized from tryptophan through the action of tryptophan hydroxylase (TPH1/TPH2) and L-amino acid decarboxylase. Neurons in the nucleus raphe medius and the nucleus raphe dorsalis send dense serotonergic projections to the hippocampus and cortex (Mowla et al., 2007). The 5-HT1A receptor (or 5-HT1AR) is highly expressed in the human hippocampus and is involved in the regulation of memory processes (Ogren et al., 2008). Patients with AD exhibit loss of cortical 5-HT neurons, reduced 5-HT levels, and the loss of dopamine (DA) neurons in the hippocampal and prefrontal areas, leading to motor disorders and cognitive decline (Morgese and Trabace, 2019). A previous study suggest that MAO inhibitors can improve cognitive dysfunction in patients with AD, while 5-HT receptor-targeted drugs show cognitive improvement in rat models of AD (Morgese and Trabace, 2019). Klaassens et al. (2019) found that selective 5-HT reuptake inhibitors (SSRIs) enhance connectivity in the DMN-precuneus/PCC, suggesting a potential role of SSRIs in memory improvement. Villemagne et al. (2013) found that escitalopram (an SSRI) may slow down the progression of AD by reducing Aβ burden, playing a crucial role in the early mechanisms of AD (Villemagne et al., 2013).

DA is synthesized from tyrosine via tyrosine hydroxylase and DOPA decarboxylase, and it modulates cortical plasticity and executive functions through D1 (excitatory) and D2 (inhibitory) receptors. DA neurons are primarily located in the ventral tegmental area (VTA) and the substantia nigra pars compacta (SNc) of the midbrain. VTA DA neurons project to the PFC, nucleus accumbens (NAc), and hippocampus, while SNc DA neurons project to the dorsal striatum. DA plays a crucial role in higher cognitive functions such as memory, learning, attention, and decision-making, and regulates excitement and pleasure. It can also influence cortical cholinergic excitability in patients with AD and in model rodents (Cole et al., 2012), affecting behavioral and psychological symptoms of dementia (BPSD), such as apathy (Šimić et al., 2017). Dysfunction in the dopaminergic system may lead to an increased prevalence of apathy in patients with AD (Mitchell et al., 2011). One possible explanation for these findings is that cholinergic neuronal projections from the laterodorsal tegmental nucleus and the pedunculopontine nucleus to the NAc can regulate DA release through M4 autoreceptors (Sarter et al., 2014). Dopaminergic influences from the SN and striatum affect the visuospatial and executive functions in patients with AD (Kang et al., 2023). Lower functional connectivity between the VTA and the parahippocampal gyrus (PHG) has been associated with agitation and irritability in patients with mild cognitive impairment and AD, whereas disruptions to sleep were associated with reduced functional connectivity between the VTA and the striatum in the same group of patients (Serra et al., 2018).

The monoaminergic system represents a compelling target for research aimed at understanding the mechanisms underlying the pathogenesis of AD and as a potential therapeutic target in the future. Studies have found that methylphenidate (MTP, which can inhibit DA reuptake), RTG (a DA agonist), and selegiline (a MAO inhibitor) are effective in improving symptoms in patients with AD (Koch et al., 2014). Several monoaminergic nuclei (locus coeruleus [LC], dorsal raphe nucleus [DRN], substantia nigra [SN]/VTA) have emerged as potentially critical to the progression of AD (Bonifazi et al., 2024).

### Intricate interrelationships among amyloid-β plaque deposition, large-scale neural network reorganization impairment, and neurotransmitter dysfunction in patients with Alzheimer’s diseases

Emerging translational evidence implicates dopaminergic dysregulation as a pivotal contributor to cognitive decline in AD (La Barbera et al., 2025). This evidence demonstrates selective degeneration of VTA dopaminergic neurons in transgenic AD models during pre-plaque pathological stages—a neurochemical derangement that precedes Aβ deposition and tau hyperphosphorylation (Nobili et al., 2017). This mesocorticolimbic DA depletion manifests through the progressive uncoupling of hippocampal-ventral striatal circuits, leading to synaptic plasticity deficits via impaired D1 receptor-mediated cAMP/PKA/CREB signaling cascades that govern dendritic spine stabilization and the maintenance of long-term potentiation (Kemppainen et al., 2000). Crucially, the spatiotemporal progression of VTA neurodegeneration correlates with the epigenetic silencing of neurotrophic factor expression and a catastrophic collapse of novelty-driven hippocampal neurogenesis, thereby mechanistically linking the erosion of dopaminergic tone to the hierarchical disintegration of memory precision and cognitive flexibility that defines early AD symptomatology. Furthermore, the presentation of unpredicted novel stimuli in recognition memory tasks triggers co-activation of the substantia nigra/VTA complex, accompanied by strengthened functional connectivity between dopaminergic midbrain projections and hippocampal CA1-subiculum networks. This interaction reflects a cooperative process where phasic DA release boosts hippocampal long-term potentiation (LTP) via D1 receptor-driven cAMP/PKA signaling pathways, thereby prioritizing novel information for consolidation (Spoleti et al., 2024). The noradrenergic and cholinergic systems, which are key neuromodulators involved in encoding memory salience and detecting novelty, undergo early and selective degeneration in AD. This degeneration is characterized by a gradual loss of NE projections from the LC to the prefrontal-hippocampal circuits, as well as atrophy of cholinergic neurons in the basal forebrain’s nucleus basalis of Meynert (Jeon et al., 2018). The combined failure of these neuromodulatory systems disrupts synaptic tagging through deficits in β1-adrenergic receptor-mediated cAMP/PKA signaling and impaired muscarinic M1 receptor-dependent theta-gamma cross-frequency coupling. This breakdown undermines the neurochemical framework essential for enhancing attention during novelty processing and memory consolidation. Importantly, tau pathology in the locus coeruleus-noradrenergic system appears at Braak stage II, before amyloid buildup, while cholinergic dysfunction is linked to disinhibition of hippocampal CA1 pyramidal neurons and abnormal hypersynchrony in the DMN. Taken together, these changes exacerbate the loss of cognitive flexibility and memory accuracy that characterize AD progression (Francis et al., 1999).

Advanced pharmaco-fMRI studies reveal that disease-modifying treatments for AD, particularly AChE inhibitors such as donepezil and uncompetitive NMDA receptor antagonists such as memantine, provide therapeutic benefits in two phases: alleviating symptoms and restoring neural circuits. Donepezil enhances cortical cholinergic signaling by selectively activating M1 muscarinic receptors, which improves theta-gamma phase-amplitude coupling between the right parahippocampal gyrus and the retrosplenial cortex and strengthens functional connectivity within the DMN (Solé-Padullés et al., 2013). In contrast, memantine reduces glutamate-induced excitotoxicity by blocking NMDA receptor channels in a voltage-dependent manner, normalizing abnormal SN hyperconnectivity and improving FPN integration through enhanced astrocyte-mediated glutamate clearance (Guo et al., 2018). These network-level modulations correlate with improved performance on episodic memory and executive function tasks, highglighting the interdependence of neurochemical signaling and large-scale network dynamics in cognitive remediation.

Recent multimodal neuroimaging evidence suggests that changes in functional connectivity networks may serve as early biomarkers, potentially appearing before Aβ plaques and tau tangles develop in AD. Notably, the cholinergic and noradrenergic neuromodulatory systems exert distinct regulatory effects on the DMN and SN, mainly through synaptic scaling and oscillatory entrainment mechanisms. Preclinical data show that ACh depletion selectively impairs DMN integrity by disrupting M1 muscarinic receptor-dependent theta-gamma cross-frequency coupling. Meanwhile, degeneration of noradrenergic neurons in the LC compromises SN coherence in the beta frequency band, which is essential for attentional salience processing. These neurotransmitter-specific network dysfunctions appear during the presymptomatic phase, emerging decades before the pathological hallmarks of amyloidogenesis and tau hyperphosphorylation.

Importantly, such early perturbations are tractable to targeted pharmacological intervention; α7 nAChR agonists, for instance, enhance DMN global efficiency in experimental models, while selective NE reuptake inhibitors demonstrate efficacy in normalizing SN metastability. This body of evidence underscores the strategic potential of neuromodulatory system restoration, positioning it as a paradigm shift towards developing disease-modifying therapies for early-stage AD. Additionally, patients with AD exhibit abnormal hyperactivity within the precuneus—a critical hub of the DMN governing self-referential cognition. Resting-state neuroimaging shows reduced low-frequency oscillatory power in this region, an electrophysiological signature exhibiting precise spatial overlap with incipient Aβ deposition and displaying an inverse relationship with hippocampal-dependent memory consolidation (Koelewijn et al., 2017). To investigate system-level changes in functional network topology, a longitudinal resting-state fMRI study followed nine patients with mild-to-moderate AD treated with oral rivastigmine over 24 weeks (with the dose increased from 1.5 mg twice daily to 12 mg once daily), alongside nine age- and sex-matched healthy controls. After treatment, patients showed measurable improvements in cognitive performance and daily living activities, accompanied by localized network reorganization characterized by increased functional connectivity in the bilateral caudate nuclei and the right superior temporal pole. Notably, global network properties such as small-worldness and modularity remained unchanged. These region-specific connectivity enhancements align with previous findings in donepezil-treated patients with AD over 3–6 months, where enhanced cholinergic transmission selectively influenced nodal centrality within limbic-cortical circuits but did not produce major alterations in overall brain network architecture.

In patients with early-stage AD confirmed to have Aβ pathology through biomarkers, targeted NIBS has been shown to enhance functional connectivity within the DMN (Pievani et al., 2017). This improvement correlates with better overall cognitive performance, driven by neuroplastic changes in the hippocampal-DMN circuitry. Neuroimaging after treatment revealed increased theta-gamma phase-amplitude coupling along the posterior cingulate-hippocampal pathway. Parallel investigations in mild AD cohorts revealed that applying NIBS to hubs in either the DMN or the SN produces comparable levels of network modulation but leads to different cognitive outcomes: stimulation targeting the DMN mainly boosts episodic memory consolidation, while SN stimulation improves attentional set-shifting. These effects correspond to the engagement of cholinergic and noradrenergic neuromodulatory systems, respectively (Li et al., 2023). Neuromodulatory techniques such as transcranial magnetic stimulation (TMS) and tDCS can normalize abnormal SN connectivity patterns, as shown by decreased functional connectivity between the anterior insula and the amygdala after intervention, alongside better scores on the Neuropsychiatric Inventory (Pini et al., 2022; Taylor et al., 2025). This therapeutic benefit, facilitated by plasticity in GABAergic interneurons and the restoration of noradrenergic activity, highlights SN dysfunction as both a marker of disease progression and a modifiable target for network-level treatments. These findings emphasize the critical role of SN synchronization in the hierarchical decline of cognitive and behavioral functions during neurodegeneration.

Growing evidence collectively highlights that abnormally large-scale brain network dynamics play a key role in understanding the underlying mechanisms of neurological disorders. Neurotransmitter systems, such as cholinergic, GABAergic, and glutamatergic signaling, critically regulate the integrity of brain connectivity by modulating synaptic plasticity and coordinating neural oscillations. These neurochemical systems not only shape the hierarchical structure of resting-state brain networks but also represent promising targets for circuit-based treatments. For example, AChE inhibitors help stabilize the default DMN, while positive allosteric modulators of GABA-A receptors can adjust hypersynchrony in the SN, effectively linking molecular dysfunctions to therapeutic strategies aimed at restoring large-scale network function.

## Neuromodulation Techniques Regulate the Function of Targeting Regions, Circuits, and Neurotransmitters

Neurostimulation techniques have gained significant attention in the treatment of AD in recent years. These techniques mainly include non-invasive methods such as rTMS, tDCS, tACS, functional near-infrared spectroscopy (fNIRS), and invasive approaches such as DBS and VNS. These methods modulate specific brain regions to restore function and slow cognitive decline. While preliminary investigations have explored NIBS-induced improvements in clinical symptomatology and cognitive performance across neurodegenerative disorders such as AD and Parkinson’s disease, existing studies have predominantly focused on symptom amelioration and regional activation patterns, with limited examination of large-scale network dynamics, particularly the reorganization of functional connectomics underlying disease progression. This suggests a critical gap in understanding how NIBS-mediated neuroplasticity reshapes hierarchical network architecture to confer therapeutic benefits (Pini et al., 2022). Emerging evidence suggests that neuromodulatory interventions in AD may exert therapeutic effects through targeted regulation of key neurocognitive networks, including the DMN, SN, CEN, and the limbic-associated Papez circuit. Anatomical targets of particular interest include the subgenual cingulate cortex (sgCC)—a hub for emotion-memory integration with dense projections to the hippocampus—and the anterior limb of the internal capsule (ALIC), a critical white matter conduit linking prefrontal cortical regions to subcortical nuclei. Stimulation of the sgCC may normalize DMN hyperconnectivity through GABAergic interneuron-mediated inhibition of glutamatergic overactivity, while ALIC-directed protocols could modulate SN-CEN cross-talk via dopaminergic mesocortical pathway potentiation, thereby restoring cognitive control over aberrant salience processing. Crucially, the sgCC-ALIC axis intersects with Aβ and tau propagation routes, offering a dual mechanism to disrupt proteinopathic spread while enhancing network resilience through activity-dependent neuroplasticity (Cheyuo et al., 2022).

### Non-invasive brain stimulations

**TMS:** TMS is a non-invasive neuromodulation method based on Faraday’s law of electromagnetic induction. Transient magnetic field pulses can induce electric currents in the cerebral cortex, modulating cortical activity. rTMS generates high-intensity pulsed magnetic fields (1.5–3.0 Tesla) capable of inducing focal eddy currents in targeted cortical laminae (depth penetration: 2–3 cm), thereby modulating the excitability of pyramidal neuron populations through depolarization of apical dendrites. The neurobiological impacts of rTMS extend beyond the immediate areas of stimulation by spreading through synaptic connections along hierarchical network pathways (Siebner et al., 2022). This process is facilitated by long-range glutamatergic projections and thalamocortical circuits. This neuromodulatory cascade restores neural coherence through homeostatic plasticity mechanisms, such as the metaplastic downregulation of NMDA receptor trafficking and activity-dependent potentiation of GABAA receptor-mediated inhibitory postsynaptic currents. Importantly, rTMS increases the release of key neurotransmitters—GABA, glutamate, DA, and ACh—in critical neurocognitive regions: the dorsolateral prefrontal cortes (DLPFC; Brodmann areas 9/46), which controls working memory and executive functions; the parietal cortex (Brodmann areas 7/40), involved in spatial attention and episodic memory retrieval; and the precuneus (PC) (Brodmann areas 7), a DMN nexus mediating self-referential processing and autobiographical memory integration (Palacino et al., 2025). The DLPFC, which is part of several neurocognitive networks related to cognition, emotional regulation, and sensory processing, is a primary target for neuromodulation. Stimulation protocols aimed directly at the DLPFC have been shown to influence episodic memory performance, potentially through its functional connections with subcortical memory systems via cortico-thalamic-hippocampal pathways. The rapid changes and high-amplitude electric field gradients (< 300 μs) induced by TMS are sufficient to depolarize the membrane potentials of cortical neurons, leading to the immediate appearance of action potentials (suprathreshold stimulation) (Peng et al., 2018). The seminal study by Koch et al. (2018) demonstrated that rTMS targeting the PC induces selective enhancement of episodic memory performance while exhibiting no detectable effects across other cognitive domains. Importantly, in patients with early-stage AD, the PC is identified as a key brain region involved in memory deficits, with growing evidence indicating that its pathological role may result from widespread network connectivity disruptions, especially within the DMN. Furthermore, multimodal transcranial magnetic stimulation combined with electroencephalography (TMS-EEG) analyses revealed altered neurophysiological patterns in patients with AD, including increased activity in PC-related neural groups, heightened beta-band oscillations, and impaired functional connectivity between the PC and mPFC nodes of the DMN. These findings suggest that frequency-specific network desynchronization could serve as an early biomarker of neurodegenerative changes (Koch et al., 2018). Another important study by Michele Maiella and colleagues involving 20 patients with mild-to-moderate AD demonstrated that neuronavigated TMS-EEG targeting the PCC—a pivotal hub of the DMN—can act as a novel neurophysiological marker for DMN integrity (Maiella et al., 2024). In healthy individuals, TMS-evoked cortical activation exhibited propagation along cingulo-temporal pathways, whereas in patients with AD, responses were limited to the stimulation site (PCC), indicative of impaired network spread. Diffusion tensor imaging revealed a robust positive relationship between fractional anisotropy (FA) of the cingulum bundle—a white matter tract connecting the PCC and MTL—and the strength of DMN functional connectivity. AD-related impairments were evident as reduced signal transmission between the PCC and frontal/temporal DMN regions. Spectral coherence analyses further revealed decreased synchronization in theta and gamma frequency bands between the PC (Brodmann area 7) and mPFC, as well as between the PC and temporal pole (Maiella et al., 2024). Notably, gamma-band desynchronization was linked to markers of synaptic dysfunction, reflecting impaired activity-dependent plasticity associated with NMDA receptor hypofunction and downregulation of brain-derived neurotrophic factor-TrkB signaling pathways (Maiella et al., 2024).

Notably, gamma-band desynchronization has been linked to markers of synaptic dysfunction, reflecting impaired activity-dependent plasticity associated with NMDA receptor hypofunction and downregulation of BDNF-TrkB signaling pathways (Maiella et al., 2024). The extended clinical investigation by Koch et al. (2025) enrolled 32 patients with mild-to-moderate AD in a 52-week randomized trial using PC-focused rTMS. Longitudinal neuropsychological assessments revealed that sustained PC-rTMS intervention significantly attenuated composite cognitive decline, preserved functional autonomy in instrumental daily activities, and mitigated the progression of neuropsychiatric symptoms. Crucially, there were no intervention-related serious adverse events (SAEs), establishing PC-rTMS as a clinically viable neuromodulatory strategy for managing early AD (Koch et al., 2025). A recent study showed that patients with AD who underwent 2 weeks of high-frequency rTMS stimulation on the left parietal lobe, which exhibits the highest functional connectivity to the hippocampus, experienced notable improvements (Wei et al., 2022). These patients had higher Mini-Mental State Examination (MMSE) scores, enhanced memory and language functions, and increased dynamic functional connectivity within the DMN. This suggests that high-frequency rTMS is an effective short-term intervention for improving cognitive function in patients with AD (Wei et al., 2022). rTMS therapy typically induces deactivation of the DMN, accompanied by reduced functional connectivity between three key DMN regions and the posterior cingulate gyrus (PCG). Changes in functional connectivity between the PCG and the left anterior cingulate gyrus correlate with improvements in auditory verbal learning and recall performance. Patients with lower baseline DMN activation exhibit heightened responsiveness to rTMS interventions. In individuals with mild cognitive impairment, targeted deactivation of hyperactivated DMN nodes effectively reverses memory deficits and may stabilize physiological DMN activation patterns (Cui et al., 2019).

**tDCS:** tDCS applies a weak, constant electric field to the brain, causing changes in the membrane potential of cells in the stimulated area, ultimately leading to excitatory or inhibitory effects (Luo et al., 2020). tDCS induces a subtle shift in neuronal membrane potential, either towards depolarization or hyperpolarization, thereby modulating the likelihood of spontaneous action potentials through subthreshold stimulation. The primary targets of tDCS include the DLPFC, temporoparietal cortex, frontal cortex, parietal cortex, temporal cortex, and language-associated Broca’s and Wernicke’s areas (Palacino et al., 2025). Luo et al. (2020) found that stimulating 6-month-old APP/PS1 transgenic mice with anodal tDCS (atDCS) improved spatial learning and memory while reducing Aβ deposition, highlighting the potential of atDCS in early-stage AD treatment (Luo et al., 2020).

In patients with AD, atDCS increases DMN connectivity, while ctDCS reduces SN connectivity (Pini et al., 2022). In another study, Hu et al. (2022) applied 4 weeks of combined rTMS-tDCS treatment at the bilateral angular gyrus (AG, P5/P6 electrode sites) to 84 patients with AD, resulting in significant improvements in their scores on the Neuropsychiatric Inventory, MMSE, and Pittsburgh Sleep Quality Index (PSQI). These findings suggest that rTMS-tDCS applied to the bilateral AG effectively improves neuropsychiatric symptoms and cognitive function in patients with AD (Hu et al., 2022). Sixty-three patients with mild cognitive impairment underwent 10 sessions of tDCS. Post-intervention analyses revealed that tDCS significantly enhanced intra-network functional connectivity within the SN and inter-network functional connectivity between the cognitive control network (CEN) and SN. In cognitively healthy older adults, tDCS has also been found to modulate both task-related and resting-state functional connectivity, correlating with improved cognitive performance. When applied during working memory tasks, tDCS increased functional connectivity within prefrontal cortical networks, with females showing significantly greater modulation of interactions between the DMN and SN compared to males— a neurobiological difference that may be due to sex-specific neurotransmitter systems (Kang et al., 2024). Subsequent research using high-definition tDCS (HD-tDCS) targeting the left DLPFC led to notable increases in temporal signal variability within key regions such as the PCC and bilateral DLPFC. This precision neuromodulation protocol yielded clinically significant outcomes, including higher scores on the Montreal Cognitive Assessment (MoCA) and positive trends in MMSE results. Quantitative analyses confirmed stimulation-induced changes in dynamic functional connectivity across eight regions of interest (ROIs), particularly affecting the middle frontal gyrus, PCC, right DLPFC, and IPL. Importantly, measures of temporal variability in the superior and middle frontal gyri—central components of the DMN—demonstrated significant associations with MoCA scores (Zhang et al., 2022), suggesting their utility as electrophysiological markers of cognitive function. These results are especially relevant given accumulating evidence of significant structural and functional deterioration of the SN throughout the AD-mild cognitive impairment spectrum, where increased regional temporal variability reflects reduced temporal stability in functional connectivity patterns (Zhang et al., 2022).

**tACS:** Compared with tDCS, tACS uses a weak alternating electric field to deliver a current that oscillates at a specific frequency and intensity with zero phase to the brain. This process promotes the synchronization of brain network oscillations, modulating brain rhythms (Herrmann et al., 2016). In a study involving 46 patients with mild AD, a 3-week intervention consisting of 30 hourly sessions of either active or sham transcranial tACS was conducted. Neuroimaging analyses after the intervention revealed that the active tACS group exhibited significant increases in the fractional amplitude of low-frequency fluctuations (fALFF) within the left middle frontal gyrus (orbital part) and the right inferior frontal gyrus (orbital part), as well as increased regional homogeneity (ReHo) in the left precentral gyrus and right middle frontal gyrus compared with baseline (Wang et al., 2024). These neurophysiological changes suggest that tACS has beneficial neuromodulatory effects on neuronal activity within prefrontal cortical regions and extended neural networks, establishing fALFF and ReHo as candidate neuroimaging biomarkers for evaluating therapeutic effectiveness in patients with AD (Wang et al., 2024). Varastegan et al. (2023) conducted a double-blind, randomized, sham-controlled parallel study involving 16 patients with subjective memory complaints (SMC), applying 6 Hz theta-frequency tACS to the mPFC. The study found that theta tACS to the mPFC enhanced event memory in individuals with SMC by modulating the activity of the frontal and temporal brain regions. These results suggest that tACS could be a promising therapeutic approach for improving memory function in SMC patients. Gamma-tACS is associated with increased cholinergic activity and enhanced network connectivity. During the late stages of AD, reduced hippocampal activation during memory encoding and persistent deactivation of the DMN are observed. tACS has been shown to improve intracortical connectivity and cholinergic transmission. Primary target regions include the left temporoparietal cortex, unilateral (left or right) temporal cortex (Dhaynaut et al., 2022), DLPFC (Kehler et al., 2020), PC (Benussi et al., 2021), and personalized regions identified through amyloid PET imaging (Palacino et al., 2025).

**tNIRS:** tNIRS is a relatively novel NIBS technique (Song et al., 2020) and a form of photobiomodulation therapy. Near-infrared (NIR) light, with wavelengths ranging from 800 nm to 2500 nm, can penetrate both soft and hard tissues, reaching deep structures within the human body at specific wavelengths (Nizamutdinov et al., 2022). tNIRS has been shown to be safe and convenient, allowing patients to undergo treatment at home (Nizamutdinov et al., 2022). The primary mechanism of tNIRS is the photobiomodulation of cellular processes, with oxygen metabolism playing a key role in various neuronal activities. As a result, tNIRS facilitates neuronal function through alterations in metabolism and hemodynamics (Song et al., 2020). Research by Song et al. (2020) suggests that 820 nm tNIRS induces a transient increase in cortical excitability at the stimulated site. In a double-blind, sham-controlled, crossover study involving 16 healthy subjects, Song et al. (2021) found that the simultaneous application of tNIRS and tDCS resulted in more pronounced cortical excitatory effects. These findings imply that tNIRS, particularly in combination with tDCS, may offer novel and potentially more effective treatment strategies in the future. In a placebo-controlled, randomized, double-blind study, Nizamutdinov et al. (2022) demonstrated that tNIRS treatment improved anxiety, depression, and cognitive function in patients with AD. Berman et al. (2017) conducted a multicenter, double-blind, randomized, placebo-controlled clinical trial using brief, repetitive 1072 nm infrared light stimulation on the cortical surface of participants. The results indicated that infrared light stimulation could improve executive function in patients with AD, though further experiments are needed to confirm changes in neuroplasticity.

**TUS:** TUS is receiving increasing attention as a novel NIBS therapy, with a growing number of human experiments being conducted. TUS offers high spatial specificity, enabling simultaneous targeting of cortical and deep brain regions (Darmani et al., 2022). TUS comprises both transcranial focused ultrasound (tFUS) and transcranial unfocused ultrasound (tUS). tFUS applies acoustic energy to specific brain regions, providing high spatial resolution and penetration depth (Jeong et al., 2022). Focused ultrasound (FUS) stimulation can target deep brain regions, which is an advantage compared with magnetic or electrical stimulation. In a preclinical study, tFUS demonstrated reductions in amyloid pathology and improvements in behavioral outcomes (Palacino et al., 2025). Eight patients with AD underwent image-guided right hippocampal tFUS immediately following intravenous administration of a microbubble ultrasound contrast agent. Participants completed MRI, ^18^F-fluorodeoxyglucose PET, and pre-/post-sonication cognitive assessments. Post-intervention analyses revealed significant improvements in immediate recall and recognition memory. FDG-PET quantification demonstrated an increased regional cerebral metabolic rate of glucose within the right hippocampus. Furthermore, the elevation of hippocampal regional cerebral metabolic rate of glucose correlated with enhanced recognition memory performance. These findings suggest that hippocampal tFUS may transiently enhance both regional glucose metabolism and memory performance in patients with AD, potentially through sonication-induced BBB modulation that facilitates neurovascular coupling restoration (Jeong et al., 2022). Sleep disorders are related to cognitive function in patients with AD, as they can exacerbate memory impairment in individuals with dementia. Therefore, enhancing the sleep status of patients with AD is significant for improving symptoms, delaying the progression of dementia, and guiding treatment (Wang et al., 2023). TUS can effectively regulate REM and NREM sleep, improving the sleep of rat models of AD with sleep disorders; however, the effects of the adjustment depend on the sleep state. TUS increases total sleep time in rat models of AD with sleep disorders and enhances the relative power in the Delta band during NREM sleep in healthy subjects (Wang et al., 2023). In 6-month-old 5×FAD transgenic and wild-type control mice, EEG electrodes and piezoelectric ceramic disk ultrasound transducers were surgically implanted on the skull surface. Park et al. (2021) found that 2 weeks of tUS decreased total Aβ_42_ and Aβ_40_ levels, particularly insoluble Aβ, in the brain cortex and hippocampus of 5×FAD mice. Additionally, tUS stimulation increased spontaneous gamma power and phase-amplitude coupling (PAC), indicative of functional improvement. In summary, tUS brain stimulation at 40 Hz may serve as a potential therapeutic modality by reducing Aβ load and improving brain connectivity (Park et al., 2021). Pyroglutamate-3 Aβ (pGlu3 Aβ), found in cerebral amyloid plaques and vascular deposits, is an N-terminally modified, pathogenic form of Aβ associated with AD. Sun et al. (2021) demonstrated that bilateral hippocampal focused ultrasound-induced BBB disruption (FUS-BBBD) led to a 5.5-fold increase in 07/2a mAb delivery to the brains compared with non-sonicated mice. This revealed a potential novel mechanism whereby the combination of 07/2a mAb with FUS-BBBD led to greater monocyte infiltration and recruitment to plaques in this AD-like model, resulting in enhanced plaque removal, preservation of synapses, and improved cognitive function without causing overt damage. Transcranial pulse stimulation (TPS) improves cognitive function in patients with AD by enhancing functional connectivity in the hippocampus, parahippocampal cortex, precuneus, and parietal cortex while activating bilateral hippocampocortical activity. Additionally, TPS alleviates depressive symptoms in AD by reducing functional connectivity between the ventromedial network (left orbitofrontal cortex) and the salience network (right anterior insula) (Chen et al., 2024). Future studies should investigate the exact neurobiological mechanisms underlying these effects.

### Invasive brain stimulation

**VNS:** The vagus nerve plays a significant role in regulating respiratory functions, heart rate, digestion, swallowing, and coughing. Activation of the vagus nerve can lead to the release of ACh at certain synaptic connections (Bonaz et al., 2016). Transauricular VNS is a NIBS method that targets the skin receptive field of the auricular branch of the vagus nerve (Ellrich, 2019). A longitudinal investigation involving 17 patients with AD who received VNS therapy over 12 months demonstrated favorable long-term tolerability, with 70.6% of participants exhibiting stabilized or improved MMSE scores compared with baseline. Serial neuropsychiatric evaluations revealed no significant deterioration in mood, behavioral symptoms, or quality-of-life metrics throughout the intervention period. CSF biomarker analyses at the 12-month endpoint showed a median reduction of 4.8% in total tau levels alongside a 5.0% increase in p-tau. These collective findings suggest that chronic VNS intervention may attenuate or delay the progression of cognitive decline in patients with AD while maintaining an acceptable safety profile (Merrill et al., 2006). A pilot study conducted by Sjögren et al. (2002) indicated a positive effect of VNS on cognition in patients with AD. Ten patients underwent implantation of a VNS device following comprehensive preoperative evaluations, including neuropsychological assessments (Alzheimer’s Disease Assessment Scale-Cognitive Subscale [ADAS-cog] and MMSE), cranial CT, and multidisciplinary medical, neurological, and psychiatric examinations, as well as lumbar puncture for CSF biomarker profiling. Longitudinal follow-ups at 3 months post-intervention revealed therapeutic responses in 7 out of 10 patients (median improvement of 3.0 points on the ADAS-cog) and 9 out of 10 patients (median improvement of 1.5 points on the MMSE) at the 3-month interval. By the 6-month endpoint, 7 patients maintained cognitive improvements on both the ADAS-cog (median Δ = 2.5 points) and MMSE (median Δ = 2.5 points). These findings provide preliminary clinical evidence supporting the potential of VNS to elicit sustained cognitive benefits in patients with AD, as quantified by standardized dementia rating scales.

**DBS:** DBS involves the implantation of electrodes to provide sustained stimulation to neural structures and is an effective long-term treatment for movement disorders such as essential tremor, Parkinson’s disease, and dystonia. The underlying premise of its therapeutic effect on many neurological and psychiatric disorders lies in the disruption of circuitry function within single or multiple neural networks (Xu and Ponce, 2018). Currently, DBS has also been found to mitigate the progression of cognitive decline in patients with AD. The first DBS study targeting patients with AD dates back to 1985 when stimulation of the left Meynert basal nucleus (NBM) was performed in a 74-year-old patient with AD. Although no improvement in memory or cognition was observed, PET scans indicated increased glucose metabolism in the ipsilateral temporal and parietal regions postoperatively (Turnbull et al., 1985). A more recent study found that tonic DBS of the thalamic nucleus reuniens can inhibit interictal epileptiform spikes that emerge during anesthesia in the CA1 and mPFC regions, thereby reducing memory damage (Shoob et al., 2023).

Laxton et al. (2020) conducted bilateral fornix DBS treatment for six patients with suspected early-stage AD (MMSE score above 20) over a duration of 12 months. They observed increased fluorodeoxyglucose uptake in the temporal and parietal lobes on PET imaging 1 month postoperatively, and MMSE scores 1 year postoperatively suggested a reduced rate of cognitive decline in five patients. Jiang et al. (2022) performed DBS targeting the NBM in eight patients with late-stage AD and found that 1 month of DBS increased the connectivity between the hippocampal network and the FPN, as well as the proportion of connections related to the parahippocampal gyrus (PHG), all of which were associated with improvements in cognitive function (Jiang et al., 2022).

## Potential Neuromodulation Strategies Targeting Region-Specific Neurotransmitter Systems in Alzheimer’s Disease

**Figure 3:**
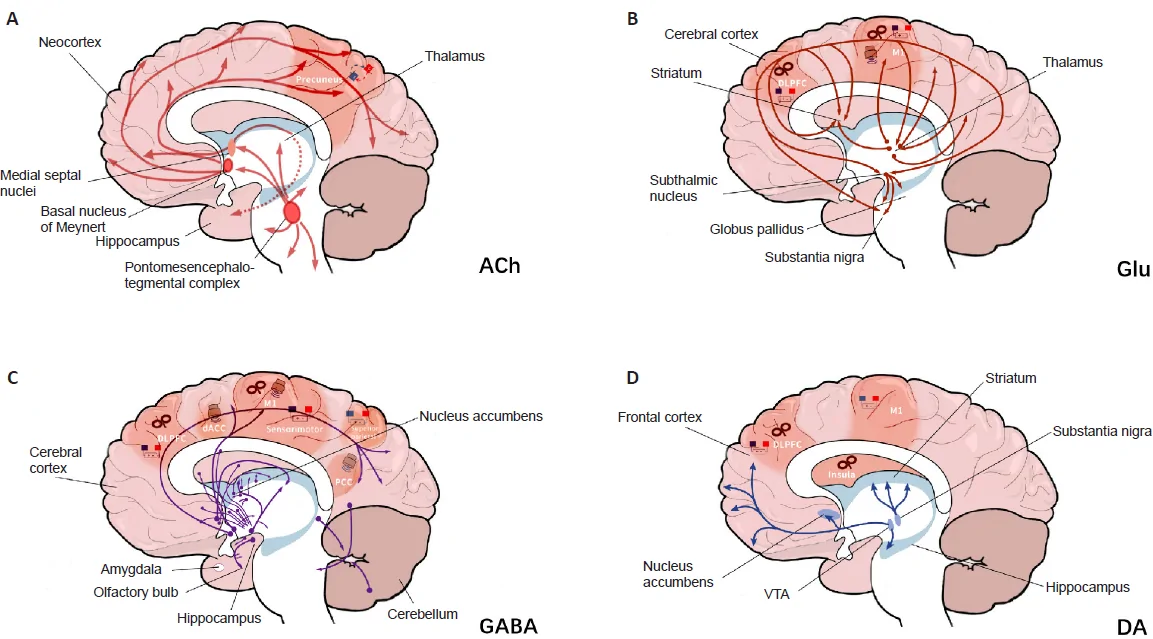
Effects of non-invasive neuromodulation techniques targeting different brain regions on various neurotransmitters. (A) Red lines denote ACh projections throughout the brain. tACS targeting the medial parietal cortex and precuneus regulates the ACh pathway. (B) Brown lines represent DA projections within the brain. TMS targeting the DLPFC and M1, tDCS targeting the DLPFC and M1, and tFUS targeting M1 regulate the glutamate pathway. (C) Purple lines indicate GABA projections across cerebral regions. TMS targeting the DLPFC and M1, tDCS targeting the right superior parietal cortex, DLPFC, M1, and left sensorimotor cortex (SM1), and tFUS targeting the dACC, left PCC, and M1 regulate the GABA pathway. (D) Blue lines depict DA projections spanning the brain. TMS targeting the bilateral insula cortex and right and left DLPFC, as well as tDCS targeting M1 and the right and left DLPFC, regulate the DA pathway. ACh: Acetylcholine; DA: dopamine; dACC: dorsal anterior cingulate cortex; DLPFC: dorsolateral prefrontal cortex; GABA: γ-aminobutyric acid; Glu: glutamate; PCC: posterior cingulate cortex; tACS: transcranial alternating current stimulation; tDCS: transcranial direct current stimulation; tFUS: transcranial focused ultrasound; TMS: transcranial magnetic stimulation; VTA: ventral tegmental area.

Neurostimulation can regulate the levels of neurotransmitters in the brain, thereby improving symptoms of AD. Below, we examine their mechanisms through the lens of neurotransmitter systems, integrating both invasive and non-invasive approaches (**Figure 3**).

### Neurostimulation techniques that regulate the cholinergic system

Neurostimulation techniques can target the medial parietal cortex, precuneus, hippocampus, and cortex to regulate the cholinergic system, not only affecting the density of ACh but also altering the levels of ChAT and the a7nAChR/Akt pathway. TMS-EEG analyses have detected cortical hyperexcitability in the precuneus, which may directly involve the degeneration of inhibitory GABAergic interneurons. Long-term cholinergic therapy with AChE inhibitors demonstrates enhanced functional connectivity between the DMN and associated hippocampal circuits (Maiella et al., 2024). tDCS has been shown to continuously improve cognitive function and memory in a rat model of AD while significantly increasing ChAT levels (Yang et al., 2019), suggesting that tDCS may have a regulatory effect on ACh levels. In a randomized, double-blind, sham stimulation-controlled, crossover pre-experimental study, Benussi et al. (2021) treated 20 participants with mild cognitive impairment and AD with a single session of 60 minutes of 40 Hz γ-tACS. The stimulation target was Pz (according to the EEG 10–20 system), covering the regions of the medial parietal cortex and precuneus. Real γ-tACS significantly improved patients’ memory performance. The study also measured cholinergic neurotransmission connectivity indirectly through short-latency afferent inhibition, revealing a restoration of cholinergic neurotransmission connectivity. VNS can induce changes in ACh, promoting a shift in the phenotype of microglial cells from pro-inflammatory to anti-inflammatory. This stimulation facilitates the degradation of Aβ by microglial cells, increases the release of neurotrophic factors, and inhibits the release of cytokines involved in inflammation, thereby exerting a protective effect (Slater and Wang, 2021). VNS, when applied to the spleen, promotes ACh production, enhances calcium uptake, and stimulates the release of alpha granules, leading to reduced bleeding and accelerated blood clotting (Bravo-Iñiguez et al., 2023). The activation of cholinergic signaling via VNS, coupled with the a7nAChR/Akt pathway, inhibits inflammation and apoptosis, providing neuroprotection against acute cerebral ischemia-reperfusion injury (Jiang et al., 2014). A study has shown that VNS can activate cholinergic and noradrenergic regulatory pathways, resulting in widespread cortical activation and increased arousal states in subcortical structures (Collins et al., 2021). Research by Bowles et al. (2022) suggested that VNS can drive motor learning through the modulation of cholinergic signaling. It has also been introduced as an alternative to invasive VNS surgeries (Kreuzer et al., 2012). In rats, transcutaneous auricular VNS improves postoperative cognitive impairment, possibly by preventing neuroinflammation, and its anti-inflammatory and neuroprotective effects may be associated with the activation of α7nAChR signaling (Cai et al., 2019). Huang et al. (2019) treated C57BL/6-Tg transgenic mice with DBS and found a significant increase in ChAT activity and a decrease in AChE activity in the hippocampus and cortex, suggesting that DBS increases ACh content in the brain. Jeong et al. (2017) conducted MS-DBS in 41 rats and found that memory impairment caused by cholinergic denervation could be improved by 5 days of stimulation before Morris water maze training, which was associated with an increase in BDNF levels. This suggests that early use of MS-DBS in the disease process may help restore spatial memory impairments.

### Neurostimulation techniques that modulate glutamate-γ-aminobutyric acid balance

Glutamate and GABA can be converted into each other. Neuromodulation techniques can effectively regulate glutamate and GABA concentrations in the M1, DLPFC, right superior parietal cortex, left primary sensorimotor cortex (SM1), dACC, and left PCC. Additionally, the potential mechanisms of gamma oscillations involve synchronous inhibitory postsynaptic potentials of GABAergic interneurons, which are related to cognitive functions (Adaikkan and Tsai, 2020). A TMS study comparing patients with AD and healthy volunteers finds that changes in GABAB and glutamate function are important factors in cognitive impairment, and that increasing degrees of dementia are associated with more significant changes in the excitability of the motor cortex (Khedr et al., 2011). A pilot study supports this point, showing that TMS increased cortical excitability and reduced inhibition, suggesting a role for asymptomatic changes in the GABAergic and cholinergic systems (Issac et al., 2013). In another study, TMS applied to stimulate the DLPFC was found to modulate the excitatory and inhibitory balance, revealing differences in the strength of GABAA and glutamate-mediated neurotransmission between older adults (> 60 years) and younger adults (18–59 years) (Noda et al., 2017). tDCS uses low-intensity continuous currents (1–2 mA) to modulate neuronal resting membrane potential, involving alterations in GABAergic and glutamatergic receptors (Palacino et al., 2025). Research by Stagg et al. (2009) demonstrated that when tDCS targets the left M1 and the contralateral supraorbital ridge, atDCS can lead to a significant reduction in GABA concentration, while cathodal tDCS (ctDCS) can decrease glutamate, which is associated with a correlated decrease in GABA. Antonenko et al., (2017) conducted magnetic resonance spectroscopy (MRS) assessments in 48 older adult individuals (24 females) stimulated with atDCS and found a decrease in GABA levels in the left SM1, a major node of the sensorimotor network. Hone-Blanchet et al. (2016) found that while atDCS targeting the left DLPFC elevated NAA in the PFC and Glx in the striatum, ctDCS targeting the right DLPFC had a similar effect. In a stud by Bunai et al. (2021), bilateral tDCS elevated GABA in the left striatum but moderately reduced it in the right striatum and left DLPFC. Research by Lengu et al. (2021) demonstrated that when tDCS is applied to the right superior parietal cortex, GABA levels increase, and the ratio of glutamate to GABA decreases. Yaakub et al. (2023) implemented a theta-burst TUS protocol on 24 healthy subjects and found that TUS reduced GABA levels in the PCC, while GABA levels in the dorsal ACC remained unaffected. Additionally, functional connectivity between the anterior and posterior cingulate cortices was enhanced. Research by Zhang et al. (2023) indicated that tFUS could modulate cortical inhibitory and excitatory processes associated with GABA and glutamate receptors, altering the levels of GABA and Glx metabolites, and thereby exerting excitatory and inhibitory regulatory effects on human motor cortical activity.

### Neurostimulation techniques that repair the monoamine system

The hippocampus, bilateral insula cortex, DLPFC, M1, VTA, MS, LC, and NAc are targets of neuromodulation aimed at repairing monoamine systems. Choung et al. (2021) applied both high-frequency (20 Hz) and low-frequency (1 Hz) rTMS to AD model mice. Behavioral assessments using the Y-maze test and novel object recognition tasks revealed cognitive recovery in a mouse model of AD under both stimulation frequencies. Neurotransmitter measurements indicated increased DA levels and DA receptor 4 expression in the hippocampus in a mouse model of AD after rTMS, with higher-frequency stimulation proving to be more effective (Choung et al., 2021). Targeting the bilateral insula cortex can decrease DA levels in the substantia nigra and the sensorimotor striatum (SMST) (Malik et al., 2018). Dorsal striatum DA release is potentiated by rTMS applied to the left DLPFC (Biernacki et al., 2023). The monoamine neurotransmitter DA mediates the neuroprotective effects of tDCS (acting on M1) in patients with AD by altering cortical neuron plasticity (Nitsche et al., 2006; Monte-Silva et al., 2010). When tDCS targets the DLPFC, it can increase extracellular DA in the striatum (Fonteneau et al., 2018). Chen et al. (2018) found that tNIR with upconversion nanoparticles can induce DA release from genetically labeled neurons in the VTA. This, in turn, induces changes in brain oscillations through the activation of inhibitory neurons in the MS and triggers memory recall. It was also found that tNIR can modulate DA release by stimulating the VTA in the mouse brain, thereby improving cognition (Chen, 2020). Evidence has shown a connection between the turnover of 5-HT and VNS (Ben-Menachem et al., 1995). Carpenter et al. (2004) discovered that VNS can alter the concentrations of NE and GABA in the CSF (Carpenter et al., 2004). VNS can induce changes in NE, promoting a shift in the phenotype of microglial cells from pro-inflammatory to anti-inflammatory. This stimulation facilitates the degradation of Aβ by microglial cells, increases the release of neurotrophic factors, and inhibits the release of cytokines involved in inflammation, thereby exerting a protective effect (Slater and Wang, 2021). A study has shown that VNS can activate noradrenergic regulatory pathways, resulting in widespread cortical activation and increased arousal states in subcortical structures (Collins et al., 2021). Shen et al., (2012) demonstrated that VNS, through the activation of the LC and beta-adrenergic receptors, induces long-term potentiation in the perforant path-CA3 region. This synaptic enhancement may underlie the clinical mechanisms of VNS in its antidepressant and anti-dementia effects (Shen et al., 2012). Ross et al. (2016) performed fornix DBS in 17 healthy domestic pigs and observed improvements in memory recall along with increased DA release in the NAc. Serotonergic pharmacotherapy correlates with increased DMN coherence linking the PC and PCC in patients with AD. Dopaminergic fibers from the mesocortical tract innervate the parietal cortex and precuneus, regions critical for DMN integration. Disruption of DMN connectivity likely results from neurochemical alterations, with reduced functional connectivity ultimately attributable to degenerative changes in mesocorticolimbic dopaminergic pathway activity (Maiella et al., 2024).

## Potential Neuromodulation Strategies for Alzheimer’s Disease Through Regulating Neurotransmitter in Specific Brain Regions

The matching of neural modulation targets and techniques represents a new approach in neural modulation therapy. DBS can regulate the concentration of ACh by acting on the hippocampus (Fontaine and Santucci, 2021), while tACS targeting the PC alters ACh levels (Maiella et al., 2024), promoting AI-guided individualization as a promising future development prospect. The hippocampus CA1/subiculum projects glutamatergic signals to the PFC, which in turn projects GABA to the core of the nucleus accumbens (NAc), subthalamic nucleus, VTA, and SNc (Thierry et al., 2000). In Tg2576AD mouse models, a decrease in glutamatergic acid projected into the core of the NAc from the CA1/subiculum damages the VTA-hippocampus-NAc pathway (Cordella et al., 2018). The lateral septal nuclei (LS) export GABAergic neurons to the MS/DBB. The MS/DBB delivers cholinergic and glutamate transmitters to hippocampus CA1 and CA3 and sends GABAergic neurons to the subiculum and CA1 of the hippocampus (Kim et al., 2022). The LC is widely projected into the cerebral cortex, including the medial PFC, orbitofrontal cortex, and ACC. This area is important for higher cognitive functions and is closely related to learning, memory, and emotion (Wiltgen et al., 2004). The thalamus and hypothalamus are key areas that connect the function of the LC and the cerebral cortex; the LC innervates the thalamus, including the intralaminar and midline nuclei, lateral habenula, and ventral anterior nuclei (Zhang et al., 1998). The LC can project noradrenaline (NA) to the hippocampus, and in 5XFAD mice, the neuroinnervation from the LC to the hippocampus is reduced. It is speculated that damage to the LC-hippocampal pathway is closely related to cognitive impairment in AD (Jeon et al., 2018). A previous study has used DBS to modulate the septo-hippocampal cholinergic pathway to restore spatial memory in patients with AD (Jeong et al., 2014). The hippocampus is recognized for its role in learning and memory and receives multiple neurotransmitters simultaneously (Kim et al., 2022). Additionally, the thalamus communicates with the hippocampus through the temporal-occipital bundle, which can inhibit hippocampal initiation network activity to reduce seizures and inhibit the thalamic occipital nucleus to prevent epileptic transmission and network activity spread. In summary, the hippocampus, as a key brain region related to memory, is an excellent target for ultrasound stimulation. Furthermore, the thalamus could also be studied as a potential target for ultrasound therapy due to its fibrous connection with the hippocampus.

## Safety Profile of Neuromodulation Techniques

Non-invasive techniques such as TMS and tDCS demonstrate high safety profiles, as they do not breach the skin or skull (Bystad et al., 2016). Short-term risks are minimal, with no significant adverse effects or only mild side effects reported under standardized protocols. Additionally, certain technologies have received FDA approval; for instance, TMS is cleared for treating refractory depression and obsessive-compulsive disorder, with rigorously validated safety (Bystad et al., 2016). In a study, six 5×FAD mice received daily 40 Hz TUS for 2 hours over 2 weeks, while six littermates underwent sham treatment. Crucially, the TUS intervention did not increase the incidence of cerebral microhemorrhage (Park et al., 2021). Overall, neuromodulation therapies exhibit relatively mild adverse effects, although further research is needed to evaluate long-term impacts, optimize technical parameters, mitigate risks, and expand clinical indications (Yang et al., 2025). Before treatment, each patient requires individualized safety and eligibility assessments by specialized clinicians.

## Current Status of Clinical Translation and Application

Contemporary investigations of neuromodulation therapeutics for AD, as evidenced by North American clinical trial registries, demonstrate robust experimental evaluation of both NIBS and IBS modalities across AD and mild cognitive impairment cohorts. These registered trials collectively report significant multidimensional benefits, including cognitive enhancement, restoration of BBB permeability, and targeted modulation of neurotransmitter systems—findings that are further substantiated by dedicated NIH funding initiatives. Current clinical evidence indicates that while IBS carries quantifiable procedural risks (with less than 5% incidence of intracranial hemorrhage or surgical site infections), NIBS exhibits an exemplary safety profile that warrants prioritized therapeutic development. Concurrently, beyond established immunotherapies (lecanemab and donanemab), the recent regulatory approval of bevacizumab (an anti-vascular endothelial growth factor monoclonal antibody) necessitates expedited yet rigorous clinical deployment to validate its neurovascular therapeutic potential (**Table 1**).

**Table 1:** Current status of clinical translation and application

Treatment	Study population	Research content	Evaluation indicators	Project number or reference
TMS	AD/MCI patients	TMS effects	Boston 30-item Naming Test and Mini-Mental Status Examination	NCT05723172
TMS	AD patients	TMS to ameliorate memory deficits	Not described	1R21AG058161-01A1
rTMS	AD patients	rTMS on the prefrontal cortex, parietal cortex, and Broca’s and Wernicke’s areas	ADAS-Cog, no adverse reactions	Nguyen et al., 2017
tDCS	AD patients	tDCS on the left temporal lobe	Neuropsychological assessment	NCT02518412
tDCS	AD/MCI patients	tDCS on DLPFC	Episodic memory performance-learning curve, GABA and Glx levels	NCT03227185
tDCS	AD patients	tDCS on left temporoparietal cortex	Improved delayed recall, no adverse reactions	Bystad et al., 2016
tACS	MCI patients	40-minute tACS for 10 consecutive daily sessions	ADAS-Cog	NCT05723172
DBS	AD patients	Bilateral fornix DBS (130 Hz)	ADAS-Cog, CDR-SB	NCT01608061
DBS	Healthy individuals	Mechanisms of memory enhancement by DBS	Not described	5R01NS084017-10
DBS	AD patients	DBS on fornix	Increased cerebral glucose metabolism with intracranial hemorrhage (1%–2%)	Lozano et al., 2016
FUS	AD patients	FUS can open BBB	BBB disruption and closure	NCT03671889
FUS	Not described	FUS for memory disorders	Not described	5R01MH133020-03
FUS	AD patients	FUS target on BBB	Enhanced potential for targeted therapeutic delivery, no adverse reactions	Rezai et al., 2021

AD: Alzheimer disease; BBB: blood–brain barrier; CDR-SB: clinical dementia rating - sum of boxes; DBS: deep brain stimulation; ADAS-Cog: Alzheimer disease assessment scale - cognitive subscale; DLPFC: dorsolateral prefrontal cortex; FUS: focused ultrasound stimulation; GABA: gamma-aminobutyric acid; Glx: glutamate + glutamine; MCI: mild cognitive impairment; rTMS: repetitive transcranial magnetic stimulation; tACS: transcranial alternating current stimulation; tDCS: transcranial direct current stimulation; TMS: transcranial magnetic stimulation.

## Limitations

This integrative review consolidates evidence elucidating how neuromodulatory interventions in AD reconfigure neurotransmitter dynamics to enhance functional connectivity and cognitive outcomes, while explicitly acknowledging inherent methodological constraints. As a narrative synthesis prioritizing thematic coherence over systematic inclusion criteria, potential selection bias warrants consideration. Substantial methodological heterogeneity—manifesting across experimental paradigms, cohort sizes, stimulation parameters, and neuropsychological assessment instruments—impedes robust cross-study comparisons and introduces mechanistically ambiguous interpretations. Emerging modalities such as tFUS necessitate rigorous validation through adequately powered randomized controlled trials to establish therapeutic efficacy and safety profiles. While demonstrating translational promise in mild cognitive impairment and early AD cohorts, the current absence of validated patient stratification biomarkers represents a fundamental methodological limitation. The predominant focus on mild-to-moderate disease stages further constrains generalizability to advanced dementia populations, where observed cognitive stabilization likely reflects compensatory neuroplastic adaptation rather than bona fide disease modification. Notwithstanding these limitations, no established consensus exists regarding optimal non-pharmacological interventions stratified by AD progression, suggesting that both mild cognitive impairment and mild-to-moderate AD cohorts warrant therapeutic trials targeting cognitive trajectory alteration. Future investigations should prioritize stage-specific efficacy stratification through targeted biomarker-guided protocols. Collectively, this analysis delineates critical advancements alongside persistent knowledge gaps, thereby charting actionable trajectories for optimizing neuromodulation paradigms and validating their translational utility within the AD therapeutic landscape.

## Conclusion

AD, a multifactorial neurodegenerative disorder exhibiting escalating global prevalence, imposes profound socioeconomic and familial burdens through its unrelenting progression. Contemporary therapeutic strategies remain predominantly pharmacologically oriented, spanning conventional cholinesterase inhibitor/memantine regimens to recently approved monoclonal antibody therapies. While established agents offer limited transient symptomatic amelioration without altering the disease trajectory—often accompanied by clinically significant adverse effects—emerging immunotherapies targeting Aβ pathology confront critical constraints: insufficient longitudinal validation of cognitive efficacy and serious safety concerns, including cerebral hemorrhage and amyloid-related imaging abnormalities. This therapeutic lacuna underscores the imperative for innovative interventions capable of safely mitigating cognitive impairment while alleviating multidimensional burdens on patients, caregivers, and healthcare infrastructures. Cognitively, higher-order processes require precisely coordinated interactions among large-scale neural networks—notably the DMN, SN, and CEN—alongside limbic circuitry, including the Papez pathway. Pathologically, the cerebral deposition of Aβ and tau proteins exhibits pathophysiological concordance with disrupted neural connectivity, potentially underpinning the emergence of cognitive deficits and neuropsychiatric manifestations in AD. Neurophysiologically, patients with AD demonstrate profound DMN architectural disintegration, starkly contrasting with the reciprocal inhibitory signaling observed in neurotypical brains, where SN activation suppresses DMN activity and vice versa.

AD manifests a characteristic neurophysiological signature featuring DMN hypoconnectivity alongside SN hypersynchrony, indicative of a metastable network equilibrium collapse fundamental to cognitive flexibility impairment. During preclinical pathogenesis, Aβ deposition preferentially targets the DMN, initiating concurrent degradation of its functional and structural connectivity. This network-selective pathogenic cascade demonstrates a topographical predilection for high-activity synaptic environments within DMN regions, suggesting activity-dependent pathogenic sequestration. Notably, functional connectivity alterations frequently precede detectable Aβ and tau pathology, serving as prodromal biomarkers of incipient neurodegeneration. The divergent neuromodulatory influence of cholinergic pathways on DMN dynamics, versus noradrenergic regulation of SN activity, further elucidates the complex neurochemical-network pathophysiology underpinning AD progression. Convergent translational evidence from clinical investigations and preclinical models indicates that precisely targeted neuromodulation interventions can effectively remodel pathological neural circuitry, demonstrating significant therapeutic potential for ameliorating cognitive deficits and affective symptoms across the AD spectrum.

Concurrently, empirical investigations establish that neuromodulatory interventions recalibrate neurochemical environments through targeted modulation of pivotal neurotransmitters—including DA, 5-HT, GABA, and glutamate—alongside their receptor expression profiles within AD-compromised neural circuitry. **Figure 3** comprehensively synthesize region-specific neuromodulation effects on neurotransmitter dynamics across these pathological networks. Such dysregulated neurotransmission constitutes a fundamental pathophysiological mechanism underlying AD pathogenesis and represents a critical therapeutic target. These techniques mediate cognitive enhancement via dual synergistic mechanisms: (1) neurotransmitter system modulation that recalibrates functional-structural network connectivity, ameliorating cognitive deficits through neurochemical rebalancing and macroscale circuit reorganization; and (2) bidirectional optimization of synaptic transmission efficiency and oscillatory coherence across distributed cortical regions. This multilevel neuromodulation synergistically restores the hierarchical architecture of cognitive processing, culminating in measurable improvements across mnemonic, attentional, and executive domains. This is the first systematic integration of the neurotransmitter regulation mechanisms of various neuromodulation techniques, with a comparison of their clinical translation potential in the treatment of AD. Future studies exploring the interference of neuromodulation techniques on key neural circuits and neurotransmitter activity in different brain regions related to AD could promote the personalized application of these technologies. This includes refining the regulatory patterns of key neurotransmitters under various neuromodulation techniques, such as how different target sites and stimulation parameters (frequency and duration) alter behavioral effects and neurotransmitter levels. Improving the safety and efficacy of research in this area will facilitate the more precise application of neuromodulation techniques in AD treatment. This, in turn, may promote the development of non-pharmacological treatment strategies for altering the course of AD and contribute to a deeper understanding of its pathogenesis. Among neuromodulatory approaches, TUS, as a relatively novel technique, offers distinct advantages: non-invasiveness, precise targeting of deep brain structures, and the capacity to induce transient BBB permeabilization. Given the hippocampus’s critical role in memory consolidation, we propose it as a promising, yet previously underrecognized, target for ultrasonic neuromodulation. Furthermore, due to the direct structural connectivity between the thalamus and hippocampus, the thalamus also merits investigation as a potential therapeutic target for ultrasound-based interventions.

## Data Availability

*Not applicable.*
